# Global Geography of Natural History Museum Specimen Holdings: A Time-Resolved Network Analysis of Mammal Collections, 1900–2020

**DOI:** 10.3897/BDJ.14.e185833

**Published:** 2026-03-26

**Authors:** Mystyn W Mills, Unna Lassiter

**Affiliations:** 1 California State University Long Beach, Long Beach, United States of America California State University Long Beach Long Beach United States of America

**Keywords:** natural history collections, museum specimens, specimen flows, biodiversity informatics, GBIF, network analysis, inequality, Gini coefficient, reciprocity, capacity building, Canidae, Felidae, Mustelidae

## Abstract

**Background**: Natural history collections are foundational infrastructure for biodiversity science, but the global geography of specimen custody — where specimens are housed relative to where they were collected — has rarely been quantified through time. Understanding these patterns is essential for assessing equitable access, providing information for capacity-building efforts and ensuring that countries of origin benefit from biodiversity research.

**Objectives**: We provide the first time-resolved, country-to-country analysis of specimen flow networks for vertebrates, using three globally distributed mammalian families (Canidae, Felidae, Mustelidae) as case studies. We ask: (1) How have regional shares of specimen holdings changed over time? (2) Have holdings become more evenly distributed across countries? (3) How have origin→holding flows shifted with respect to regional retention and reciprocity? (4) Do the three families exhibit distinct curation profiles?

**Methods**: We analysed 253,131 preserved-specimen records from GBIF spanning 1900–2020, aggregated into four approximately equal temporal periods (1900-1929, 1930-1959, 1960-1989, 1990-2020). We constructed directed, weighted country-to-country flow networks for each family × time period combination and computed network metrics (size, complexity, density, reciprocity, connectivity) and inequality metrics (Gini coefficient). We visualised geographic patterns using network maps on Robinson projections and assessed temporal trends using statistical models.

**Results**: Specimen holdings are highly concentrated: the United States holds 55% of all specimens and the top 10 countries account for 90%. Inequality follows a U-shaped trajectory: high concentration in early periods (Gini 0.85–0.89 in 1900-1929), democratisation by the 1960-1989 period (Gini 0.77–0.89), then re-concentration in 1990-2020 (Gini 0.81–0.86). Specimen flow networks underwent simplification, contracting from 100–111 participating countries and 207–262 flows in the 1930-1959 period to only 75–84 countries and 115–160 flows in 1990-2020 — a 23–29% reduction in network size and 39–56% reduction in complexity. Despite simplification, networks remain dense and cohesive amongst remaining participants. However, reciprocity (bidirectional exchange) is low (mean 0.075 or 7.5% in recent periods), indicating predominantly unidirectional flows from biodiversity-rich regions to museum-rich nations. These patterns are broadly consistent across Canidae, Felidae and Mustelidae, though Felidae exhibits less inequality rebound, possibly reflecting conservation-driven capacity building.

**Conclusions**: Our findings reveal persistent geographic imbalances in specimen custody, characterised by concentration in wealthy nations, low reciprocity and network contraction over time. While some patterns may reflect digitisation lags, genuine reductions in the breadth of global collecting and shifts towards extractive (non-reciprocal) flow structures are evident. These imbalances create structural inequities in research access, taxonomic expertise and capacity for biodiversity science in countries of origin. Addressing these challenges requires systemic interventions: capacity building in under-represented countries, collaborative frameworks that ensure reciprocity and benefit-sharing, improved digital access, policy reforms to streamline permits without compromising sovereignty and sustained funding for museum science and taxonomy. This study provides a quantitative baseline for monitoring progress towards more equitable specimen curation and offers a reproducible framework applicable to other taxa and regions.

## Introduction

Natural history collections are foundational research infrastructures for biodiversity science. They support taxonomy and systematics, enable reconstructions of ecological and evolutionary change and anchor monitoring and conservation planning (Lane 1996, Suarez and Tsutsui 2004, Pyke and Ehrlich 2010, Lendemer et al. 2019). While digitiation initiatives now make specimen metadata broadly discoverable through aggregators like the Global Biodiversity Information Facility (GBIF) and iDigBio, the physical geography of collections remains decisive for those who can access and steward material specimens. Understanding where specimens are housed relative to where they were collected is therefore essential for equitable access, curatorial capacity, partnership building and policy (Chakrabarty 2009, McLean et al. 2015, Malaney and Cook 2018, Byrne 2023).

Today, the largest natural history museums and universities with extensive faunal and floral holdings remain concentrated in countries of the Global North. While many now focus more on their local environments and display regional species, their collections remain encyclopaedic and global in scope — a legacy of colonial opportunism, nationalistic competition and centuries of scientific collecting (Chakrabarty 2009). These historical trajectories moved specimens from their points of origin to centralised Western institutions, yet contemporary flows show limited reciprocity or return, despite legal frameworks like the Nagoya Protocol and growing calls for decolonisation of museum collections (Watanabe 2017, Miriti et al. 2022). The concentration of specimens in Western institutions has profound consequences for global science: it shapes research agendas, limits local capacity building and perpetuates inequitable access to biodiversity data (Waterton 2002, Malaney and Cook 2018). While digitisation initiatives aim to democratise access by making collection data globally available online, they cannot fully address the persistence of physical specimens in distant repositories, nor do they resolve questions of repatriation, local stewardship or the development of research infrastructure in biodiversity-rich, but collection-poor regions (Vollmar et al. 2010).

Despite the growth of aggregated record portals, the "geography of curation" — the spatial relationship between collection origins and holding institutions — has rarely been quantified at global scale. Herbaria have received the most comprehensive treatment, with multiple studies documenting strong imbalances: specimens from biodiversity-rich tropical countries are disproportionately housed in institutions in Europe and North America (Park et al. 2023). Vertebrate collections are less examined and, critically, most prior work treats collections as static snapshots, obscuring temporal dynamics. Country-to-country analyses typically focus on single institutions or regions; taxon-specific comparisons across multiple families are rare; and reproducible, decadal, global views for vertebrates are limited. We lack time-resolved analyses showing how origin-holding flows evolved across decades as decolonisation, conservation regimes, digitisation priorities and funding landscapes shifted. Without temporal baselines, we cannot assess whether current imbalances are worsening, stabilising or improving, nor identify periods when interventions have succeeded or failed.

Here, we provide, to our knowledge, the first time-resolved, country-to-country assessment of specimen flows for vertebrates, analysing four approximately equal 30-year periods spanning 1900–2020 (periods: 1900–1929, 1930–1959, 1960–1989, 1990–2020; the last period is 31 years). We focus on three mammal families — Canidae (dogs, wolves, foxes; approximately 36 species), Felidae (cats; approximately 41 species) and Mustelidae (weasels, otters, badgers; approximately 70 species) — as case studies. We selected these families because they: (1) are globally distributed across diverse biomes; (2) include species of high conservation concern (many listed on the IUCN Red List) and are of historical economic importance (e.g. the mustelid fur trade); (3) have substantial museum representation spanning more than a century of collecting and (4) vary in capture opportunity, research interest and species charisma in ways that may influence collection priorities and patterns. Together, they provide a broad lens on global patterns, while allowing assessment of taxon-specific variation.

Using GBIF preserved-specimen records, we contrast collection countries (origins) with holding countries (institutions) and construct directed, weighted country-to-country flow networks for each family × period. We compute standard network metrics (size, complexity, density, reciprocity, connectivity) to characterise structure and change and measure inequality in country holdings using the Gini coefficient. We pair descriptive network visualisations with statistical tests to quantify temporal trends and family differences. Our fully reproducible R workflow (GitHub) enables application to other taxa, regions or time periods and all derived datasets are archived on Zenodo.

We ask three questions:

**Inequality trends**: Has the concentration of specimen holdings decreased over time as museums proliferated worldwide or has inequality persisted, despite expanding global infrastructure?

**Flow patterns**: Have origin→holding flows shifted towards greater regional retention and reciprocity over time or does unidirectional transfer from biodiversity-rich to museum-rich nations persist?

**Family-specific patterns**: Do Canidae, Felidae and Mustelidae exhibit distinct curation trajectories or do they follow common patterns reflecting system-wide forces?

By delivering a time-resolved, family-level, country-to-country analysis of vertebrate collection flows, we provide a quantitative baseline for monitoring geographic equity, providing information for capacity-building investments, evaluating policy interventions such as the Nagoya Protocol and assessing progress towards more collaborative and reciprocal specimen stewardship. Our framework reveals temporal trajectories that can attest to how modern collections were formed, what trends changed and when and what differences persist across regions, families and collection periods. Understanding the historical geography of specimen flows is essential for building a more equitable future for biodiversity science, one that supports the development of local museums as research nodes, strengthens North-South partnerships in museum professionalism and stems brain drain by enabling regionally-based biodiversity science.

## Material and methods

**Data acquisition and filtering**

We obtained preserved‑specimen occurrence records for Canidae, Felidae and Mustelidae from the Global Biodiversity Information Facility (GBIF) using the rgbif R package (version 3.7.7) in October 2025. Queries targeted Darwin Core basisOfRecord = "PreservedSpecimen" and returned records with family assignments in the three focal groups.

Initial queries returned 312,456 specimen records. We applied the following filters to improve data quality and analytical tractability:

1. **Basis of record**: retain only preserved specimens; exclude observations, living specimens and fossils;
2. **Country fields**: require both a collection (origin) country and a holding (institution) country. Origins were taken from dwc:country/dwc:countryCode and treated as the country in which the specimen was collected, regardless of whether the individual originated from a wild or captive population (e.g. zoo animals). Holding country was inferred from dwc:institutionCode (and, where present, dwc:ownerInstitutionCode) by cross-referencing the Global Registry of Scientific Collections (GRSciColl). Although some records include fields, such as establishmentMeans that can indicate captive origin, these annotations are incomplete and inconsistent across institutions and time and we, therefore, did not filter them;
3. **Temporal extent**: retain records with collection dates 1900–2020 (inclusive); exclude undated and pre‑1900 material to focus on the modern museum era;
4. **Specimen counts**: retain records with occurrenceStatus = "present" and **non‑zero** counts (i.e. individualCount ≥ 1; when missing, treat as a single specimen);
5. **Validation**: exclude records with missing, ambiguous or invalid country codes.

After filtering, the dataset comprised 253,131 records. We emphasise that GBIF reflects digitised and shared holdings rather than complete institutional inventories; patterns may be affected by digitisation priorities, data‑sharing practices and lags in cataloguing, especially for recent material.

**Country standardisation and temporal binning**

We standardised country names to ISO 3166‑1 alpha‑3 codes using the countrycode R package (version 1.5.0), with manual disambiguation where needed (e.g. COD vs. COG). For each specimen we extracted: (i) the collection country (origin) and (ii) the holding country (destination) inferred via GRSciColl mapping from institution identifiers.

We aggregated specimens into four approximately 30 year time bins, based on collection date:

- **1900-1929** (30 years): Colonial era and early systematic collecting expeditions;
- **1930-1959** (30 years): World War II, post-war museum expansion and intensified global surveys;
- **1960-1989** (30 years): Decolonisation, Cold War scientific exchanges and emergence of conservation biology;
- **1990-2020** (31 years): Digital era, biodiversity crisis recognition and implementation of the Nagoya Protocol (2014).

The 30-year window represents approximately one scientific generation, capturing institutional continuity, while allowing detection of paradigm shifts. We acknowledge that the most recent period (1990-2020) may be subject to greater digitisation lag, as recently collected specimens may not yet be fully catalogued in GBIF. All analyses were conducted separately for each family × period combination, yielding 12 analytical slices (3 families × 4 time periods).

Note on domestic flows. Specimens collected and held in the same country (self‑loops) were excluded from network topology calculations, but retained when computing holdings inequality.

**Network construction and visualisation**

For each analytical slice, we constructed a directed, weighted network in which nodes are countries and edges represent flows from collection (origin) to holding (destination) countries. Edge weights equal the number of specimens in a given origin→destination pair. Self‑loops were excluded from topology metrics.

We visualised networks on a Robinson world projection using Natural Earth country polygons (public domain; scale “medium”, version 5.1.1) via rnaturalearth (version 0.3.4). Country points were placed using sf::st_point_on_surface() to ensure centroids fall within complex geometries. To reduce clutter while preserving major patterns, maps display the top 200 edges by weight per period; edge thickness and alpha scale with weight. Node size depicts in‑strength (total specimens held) and node colour depicts net balance (in−out)/(in+out)(\text{in} - \text{out})/(\text{in} + \text{out})(in−out)/(in+out), ranging from −1 (pure exporter) to +1 (pure importer). Top 10 countries by in‑strength were labelled using ggrepel (version 0.9.4).

Maps and plots were generated using ggplot2 (version 3.4.4) and patchwork (version 1.1.3) in R (version 4.3.1). All cartographic operations used the sf package (version 1.0-14).

**Network metrics**

We computed six network‑level metrics for each slice using **igraph** (version 1.5.1):

- **Network size (nodes)**: number of countries with ≥ 1 **international** in‑ or out‑edge (no isolates);
- **Network complexity (edges)**: number of **unique directed** origin→destination pairs with non‑zero weight;
- **Density**: realised fraction of possible directed edges, edges/[nodes(nodes−1)]\text{edges} / [\text{nodes}(\text{nodes} - 1)]edges/[nodes(nodes−1)] (range 0–1);
- **Reciprocity**: proportion of edges that are part of **mutual pairs**, computed with igraph::reciprocity() (default mode), i.e. the fraction of existing edges that have the reverse edge. Values near 0 indicate unidirectional flows; values near 1 indicate bidirectional exchange;
- **Giant component fraction**: share of countries in the **largest weakly connected component** (computed on the underlying undirected graph), i.e., max⁡(component size)/nodes\max(\text{component size}) / \text{nodes}max(component size)/nodes;
- **Modularity**: community structure via the **Louvain** algorithm on the **undirected, weight‑summed** projection (as.undirected(mode = "collapse", edge.attr.comb = list(weight = "sum"))), where higher values indicate stronger division into sub‑networks.

**Inequality metrics**

We quantified inequality in specimen holdings using the Gini coefficient, a standard measure of distributional inequality borrowed from economics. The Gini coefficient ranges from 0 (perfect equality: all countries hold equal numbers of specimens) to 1 (perfect inequality: one country holds all specimens). We calculated Gini coefficients using the ineq::Gini() function separately for each family × period combination, based on the distribution of total holdings (in-strength, including domestic specimens) across all countries with non-zero holdings.

Importantly, we computed Gini coefficients only for countries actively holding specimens in each slice, excluding countries with zero holdings in that slice. This approach measures inequality amongst participants rather than treating non-participation as zero holdings, which would artificially inflate inequality. This decision reflects our focus on the distribution of curation responsibility amongst countries engaged in specimen stewardship.

**Statistical analyses**

To assess temporal trends, we modelled metrics against period mid-point (treated as a continuous predictor: 1915, 1945, 1975, 2005). For Gini, we fit linear and quadratic models and compared them via ANOVA to test for non‑monotonic (U‑shaped) patterns. For nodes, edges, density, reciprocity and giant component fraction, we fit linear models to estimate change per period. We report slope estimates, standard errors and *p*‑values; given four time points per family, trend results are interpreted cautiously.

Family differences were evaluated with linear mixed‑effects models (lme4::lmer), treating family as a fixed effect and period as a random effect to account for repeated measures across time. Estimated marginal means (emmeans version 1.8.9) provided pairwise comparisons with Tukey adjustments.

We examined coupling amongst metrics using Pearson correlations and a linear model predicting edges from nodes. Early (1900–1929, 1930–1959) vs. recent (1960–1989, 1990–2020) periods were compared with Welch’s *t* tests.

Sensitivity analyses evaluated robustness to: (i) alternative minimum edge‑weight thresholds (e.g. excluding flows < 5 or < 10); (ii) alternative Gini definitions (including vs. excluding zero‑holding countries) and (iii) alternative temporal binning. Qualitative conclusions were unchanged (see Supplementary Materials). All analyses used R 4.3.1; significance was set at α = 0.05, with exact *p*‑values reported for *p* ≥ 0.001 and “< 0.001” otherwise.

**Top importers and exporters**

For each family × period, we identified the top five net importers (in−out)(\text{in} - \text{out})(in−out) and top five net exporters. We report both raw net counts and a normalised net balance (in−out)/(in+out)(\text{in} - \text{out})/(\text{in} + \text{out})(in−out)/(in+out), which ranges from −1 (pure exporter) to +1 (pure importer) and is independent of total flow magnitude.

## Data resources

All analyses were implemented in R Markdown with version‑pinned packages: dplyr 1.1.3, tidyr 1.3.0, ggplot2 3.4.4, sf 1.0‑14, igraph 1.5.1, ineq 0.2‑13, patchwork 1.1.3, scales 1.2.1 and rgbif 3.7.7. Complete, executable code (data acquisition, cleaning, analysis and figures) is available at https://github.com/wildrlab/MammalMuseumHoldings. Derived datasets (cleaned flows, network metrics, Gini coefficients) are archived at Zenodo (https://doi.org/10.5281/zenodo.18224323). GBIF Occurrence Download DOI(s) and query parameters are listed in the repository README and cited in the manuscript’s *Data resources* section. The workflow is fully reproducible from raw GBIF downloads to final figures.

## Results

**Dataset overview**

Our analysis comprised 253,131 preserved-specimen records from GBIF spanning 1900–2020, representing three mammal families: Canidae (n = 94,401 specimens; 37%), Felidae (n = 41,113; 16%) and Mustelidae (n = 117,617; 46%). After filtering for valid collection‑country and holding‑country metadata, specimens originated from 178 distinct countries, but are currently held by institutions in 44 countries — a roughly 4:1 source‑to‑repository ratio indicating strong concentration of curation capacity. Across periods, the dataset captures 115–262 unique country‑to‑country flows, reflecting substantial variation in network complexity over time.

Records were aggregated into four approximately equal 30‑year periods (last period is 31 years): 1900–1929, 1930–1959, 1960–1989 and 1990–2020. Specimen counts vary across periods, with 1960–1989 containing the most digitised specimens and 1990–2020 fewer, likely reflecting digitisation lags for recent material as well as changing collecting and sharing practices. Mustelidae constitutes the largest share of records (46 %), consistent with its higher species richness and broad latitudinal distribution. Table 1 provides a summary of specimens, network metrics and inequality trends across families and periods.

**Geographic concentration of holdings**

**Figure 1.**
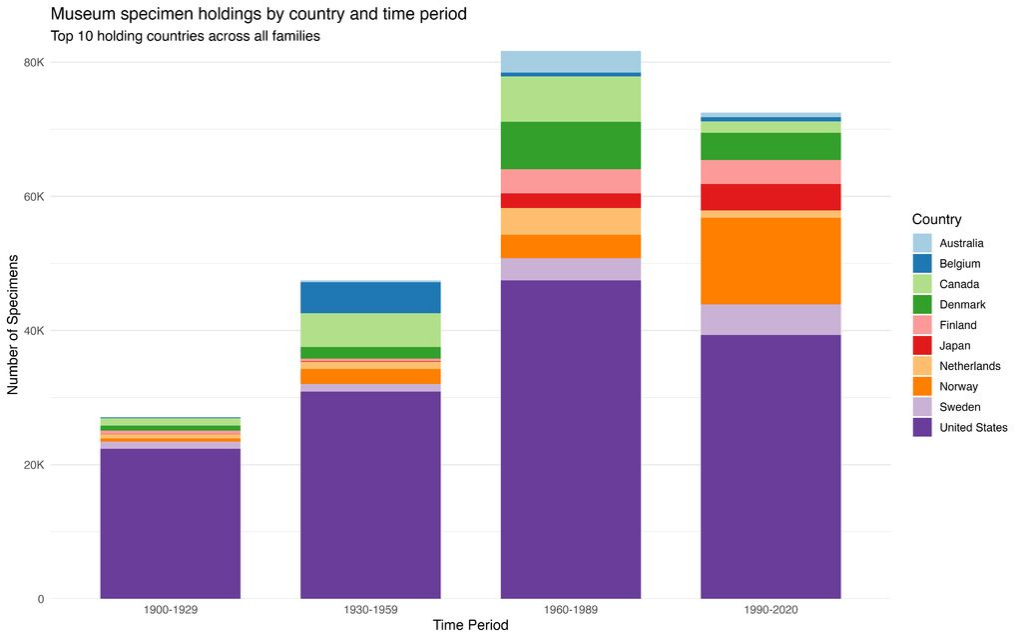
Museum specimen holdings by country and time period. Specimen holdings for the ten countries with the largest collections of Canidae, Mustelidae and Felidae across four 30-year periods (1900–1929, 1930–1959, 1960–1989, 1990–2020), based on GBIF preserved-specimen records. Each bar represents total digitised holdings per period, with countries shown as coloured segments. The United States dominates all periods, consistently holding ~ 50% of specimens. Other major holders include Norway, Sweden, Canada, Denmark, Finland, Japan, the Netherlands, Belgium and Australia. Holdings peak in 1960–1989 (~ 80,000 specimens), then decline in 1990–2020 (~ 72,000 specimens). The ten countries shown here account for ~ 89-90% of all specimens; additional countries (e.g. France, Mexico, Spain and the United Kingdom) enter the top-10 in some decades, but are not shown here for clarity.

Specimen holdings are concentrated in a small number of countries, with the United States dominating across all families and periods (Fig. 1). Over the entire study window, U.S. institutions hold roughly half of all specimens and the top 10 holding countries account for ~ 89–90%. Other major repository nations in our GBIF-derived dataset include Norway, Canada, Denmark, Sweden, Finland, the Netherlands, Japan, Belgium, Germany and Australia. Decadal top-10 lists show a persistent core of holding countries: the United States, Canada, Denmark, Norway and Sweden appearing amongst the ten largest holders from 1900 to 2020. In contrast, several countries are only transiently prominent — for example, France and Mexico rank amongst the top holders in early twentieth-century decades, but drop out of the top-10 later, whereas Japan, Spain and the United Kingdom only enter the top-10 in the late twentieth century or after 2000. Notably, the United Kingdom is relatively under-represented in our GBIF-derived sample for these families compared with its known historical collection strength. This may reflect differences specific to these families and/or in digitisation and data-mobilisation practices — many UK carnivore holdings may not yet be fully visible in GBIF — rather than a true scarcity of physical specimens. Our rankings should, therefore, be interpreted as the geography of GBIF-visible holdings rather than a complete census of global physical collections. Rankings also shift slightly by family; for example, Norway contributes proportionally more Mustelidae holdings.

**Evolution of specimen flow networks**

**Figure 2.**
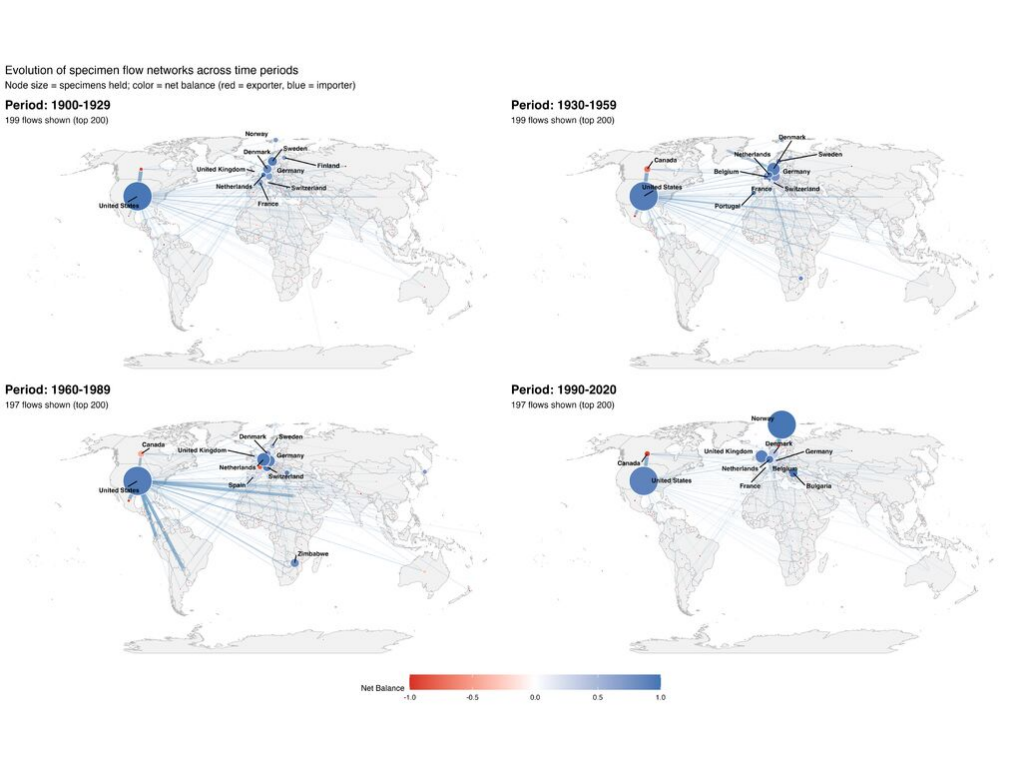
Specimen flow networks by period (families combined). Directed, weighted origin→holding flows are drawn on a Robinson projection. Node size = total in‑strength (specimens held). Node colour = net balance (in−out)/(in+out)(\text{in} - \text{out})/(\text{in} + \text{out})(in−out)/(in+out) from −1 (net exporter) to +1 (net importer). Edge thickness/alpha scale with flow weight. For legibility, plots show the top 200 flows per period (or all flows if fewer).

Country‑to‑country flow maps reveal clear temporal change in global patterns (Fig. 2). Early and mid‑century periods show dense networks with prominent hubs in North America (especially the United States) and Europe (notably Scandinavia, the Netherlands, Belgium and the United Kingdom). Many countries in Africa, Latin America and Asia appear primarily as sources, with relatively small nodes. By 1990–2020, maps show fewer active source countries, sparser edges and increased centralisation around a smaller set of hubs; Norway emerges as a major holder in the most recent period. Variation in country-to-country flow by family can be seen in Suppl. materials 1, 2, 3.

Specimen holdings are concentrated in a small number of countries, with the United States dominating across all families and periods (Fig. 1). Over the entire study window, U.S. institutions hold roughly half of all specimens and the top 10 holding countries account for ~ 89–90%. Other major repository nations in our GBIF-derived dataset include Norway, Canada, Denmark, Sweden, Finland, the Netherlands, Japan, Belgium, Germany and Australia. Decadal top-10 lists show a persistent core of holding countries: the United States, Canada, Denmark, Norway and Sweden appearing amongst the ten largest holders from 1900 to 2020. In contrast, several countries are only transiently prominent— for example, France and Mexico rank amongst the top holders in early twentieth-century decades, but drop out of the top-10 later, whereas Japan, Spain and the United Kingdom only enter the top-10 in the late twentieth century or after 2000. Notably, the United Kingdom is relatively under-represented in our GBIF-derived sample for these families compared with its known historical collection strength. This may reflect differences specific to these families and/or in digitisation and data-mobiliation practices— many UK carnivore holdings may not yet be fully visible in GBIF — rather than a true scarcity of physical specimens. Our rankings should, therefore, be interpreted as the geography of GBIF-visible holdings rather than a complete census of global physical collections. Rankings also shift slightly by family; for example, Norway contributes proportionally more Mustelidae holdings.

**Network size and complexity declined sharply over time**

**Figure 3.**
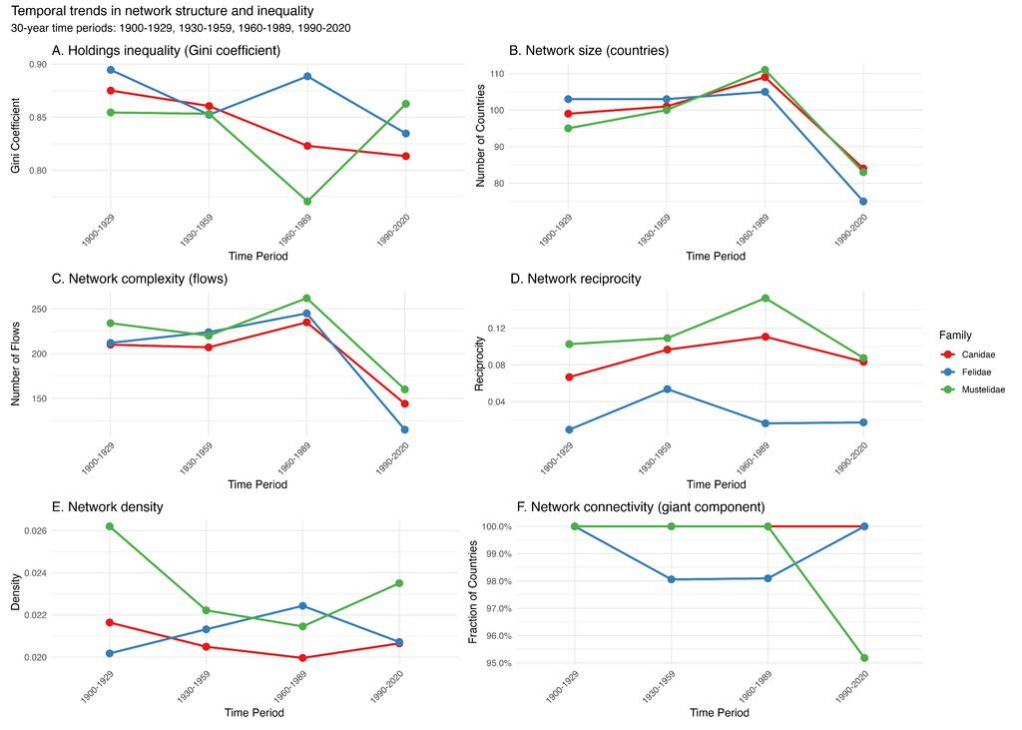
Temporal trends in network structure and inequality. Six-panel figure showing temporal changes in specimen flow network metrics for Canidae (red), Felidae (blue) and Mustelidae (green) across four 30-year periods (1900-1929, 1930-1959, 1960-1989, 1990-2020). (A) Holdings inequality (Gini coefficient): All families begin with high inequality (0.85-0.89). Mustelidae declined initially, but rebounded sharply in 1990-2020. Canidae showed a more gradual, sustained decline that levelled off in recent decades. Felidae exhibited the steepest and most consistent decline throughout the study period. (B) Network size (countries): All families peak at 100-111 countries in mid-century, then decline to 75-84 countries by 1990-2020 (23-29% reduction). (C) Network complexity (flows): Peak at 207-262 flows in 1930-1959, collapse to 115-160 in 1990-2020 (39-56% decline). (D) Reciprocity: Remains very low throughout (< 0.15), with mean 0.075 (7.5%) indicating predominantly unidirectional flows. (E) Density: Complex patterns with modest variation (0.020-0.026). (F) Connectivity (giant component): Near-complete connectivity maintained (> 95%) despite network contraction.

Quantitative metrics corroborate visual patterns (Fig. 3B–C). Participating countries peaked at ~ 100–111 in 1930–1959, then declined to ~ 105–110 in 1960–1989 and ~ 75–83 in 1990–2020 — a 25–32 % reduction over the last two periods. Linear models indicate significant negative trends across families (*p* < 0.001; *R*² > 0.95), suggesting system‑level contraction rather than family‑specific effects. Flow complexity (directed edges) was already high in 1900–1929 (207–240), peaked in 1930–1959 (234–262), declined in 1960–1989 (209–238) and fell to 115–160 in 1990–2020 (a 39–56 % drop from peak). The strong correlation between nodes and edges (*R*² > 0.95) implies that country dropout eliminates multiple connections, amplifying simplification. The edges‑to‑nodes ratio remained ~ 2.1–2.4 through mid‑century and decreased slightly in 1990–2020 for some families.

Network complexity, measured as the number of unique country-to-country flows (directed edges), followed a similar, but more dramatic trajectory. Flow complexity was already high in 1900-1929 (207–240 edges), peaked in 1930-1959 (234–262 edges), declined moderately in 1960-1989 (209–238 edges), then collapsed to only 115–160 edges in 1990-2020 — representing a 39–56% reduction from peak complexity. Statistical tests confirm significant declines across all families, with loss rates averaging approximately 70–75 edges per 30-year period (p < 0.001, R² > 0.94). The strong correlation between network size (nodes) and complexity (edges) across all slices (R² > 0.95, p < 0.001) indicates that countries dropping out of the network also eliminate multiple flows, amplifying the simplification effect.

The ratio of edges to nodes remained relatively stable at ~ 2.1–2.4 across early and mid-century periods, suggesting that participating countries maintain a consistent average number of exchange partners. However, the 1990-2020 period shows this ratio declining slightly for some families, indicating that network simplification involves both country dropout and reduction in connections per country. Comparing early periods (1900-1929, 1930-1959) vs. recent periods (1960-1989, 1990-2020) using Welch's t-tests confirms dramatic reductions in both network size and complexity (p < 0.001 for both metrics).

While the recent period's shorter temporal span contributes to reduced absolute counts, the decline in *rate* of network activity is genuine: even accounting for time-span differences, the 1990-2020 period shows substantially lower flow generation per year compared to mid-century peaks. These patterns suggest a real reduction in the geographic breadth of specimen-based fieldwork and international museum exchange, compounded, but not fully explained, by digitisation lags.

**Inequality decreased mid-century, then partially rebounded**

Holdings inequality (Gini coefficient) was very high in 1900–1929 (0.85–0.89) and remained high in 1930–1959 (0.85–0.87). By 1960–1989, all three families showed declines, with Felidae reaching the lowest inequality (0.77). In 1990–2020, however, family-specific trajectories diverged: Mustelidae rebounded to 0.86 (near initial levels), Canidae showed a moderate sustained decline to 0.81 and Felidae exhibited the steepest decline to 0.84. This U-shaped pattern in Mustelidae contrasts with the more linear declines in Canidae and Felidae. Two-way ANOVA on Gini values (family × period) indicates significant main effects of family and period (both p < 0.01), confirming that inequality differs significantly amongst time periods and amongst families, with a marginal interaction (p = 0.09).

**Density and connectivity**

Despite fewer nodes and edges in recent decades, density remained stable or increased slightly in most families (~ 0.020–0.023), consistent with a core‑densification effect in smaller networks (strong negative size–density correlation for Felidae). The giant component encompassed 95–100 % of participating countries across periods (mean 97.8 % ± 3.1 %), indicating that contraction primarily removed peripheral participants rather than fragmenting the core.

**Reciprocity is uniformly low**

Reciprocity (fraction of bidirectional pairs) was low overall and varied modestly by family: Felidae near zero (0.01–0.05), Canidae slightly higher in early/mid‑century (0.07–0.11) with a small decline by 1990–2020 (~ 0.08) and Mustelidae peaking at ~ 0.11 in 1930–1959 before converging near 0.08 in the most recent period. Mean reciprocity across families/periods was ~ 0.066, indicating that fewer than 7 % of country‑pairs show bidirectional exchange. Temporal trends are weak relative to the consistently low baseline.

**Networks remain cohesive despite fragmentation**

Despite substantial reductions in size and complexity, specimen flow networks maintained high connectivity throughout the study period (Fig. 3F). The giant component — the largest set of countries linked by direct or indirect specimen flows — comprised 95–100% of all participating countries in most periods for all three families. This near-complete connectivity indicates that, even as networks contracted, they did so in a way that largely preserved overall cohesion rather than fragmenting into disconnected sub-networks.

Notably, Mustelidae shows a sharp decline in giant component fraction in the 1990-2020 period (to ~ 95%), suggesting emerging fragmentation with small numbers of countries operating in isolation from the main network. Canidae and Felidae maintain very high connectivity (> 99%) even in recent periods. Statistical comparisons of early vs. recent periods show no significant change in connectivity for most families (p > 0.05), confirming that network cohesion has remained robust despite dramatic changes in size and complexity.

The vast majority of specimen-holding countries remain connected through chains of flows, ensuring that specimens from most source countries could theoretically be accessed through collaborations with any member of the network. This connectivity has important implications for research access and taxonomic expertise, as it suggests that researchers can potentially reach specimens from diverse source countries through partnerships with a relatively small set of hub institutions. The mean giant component fraction across all families and periods is 97.8% ± 3.1%, indicating near-universal connectivity within active networks.

**Regional and temporal imbalances in net flows**

**Figure 4.**
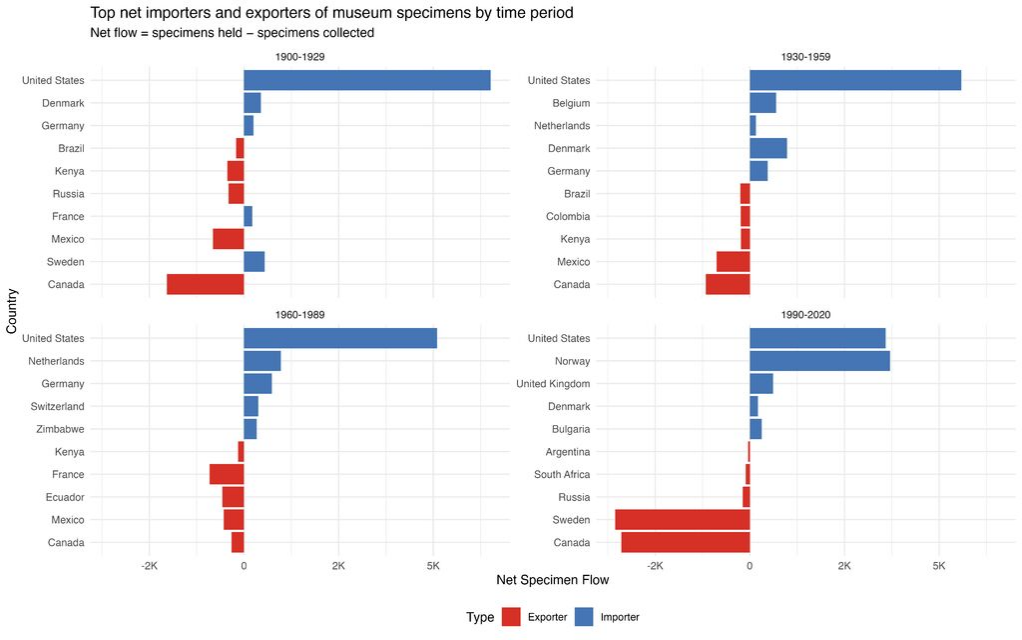
Top net importers and exporters of museum specimens by time period. Four-panel figure showing net specimen flows for all three families combined, with each panel representing one 30-year period (1900-1929, 1930-1959, 1960-1989, 1990-2020). Each panel displays top five net importers (blue bars, positive values extending right) and top five net exporters (red bars, negative values extending left). Net flow = specimens held minus specimens collected. The United States consistently dominates as top importer across all periods. Other major importers include Denmark, Germany, Netherlands, Belgium and notably Norway (large increase in net imports in 1990-2020). Top exporters include African nations (Kenya, South Africa), Latin American countries (Mexico, Brazil, Colombia, Ecuador, Argentina) and Canada. The persistence of the same countries as exporters across decades, coupled with consistent Northern importers, demonstrates sustained North-South imbalances in specimen custody.

Net‑balance profiles emphasise persistent geographic asymmetries (Fig. 4). The United States is the top net importer in all periods for all families. Other recurring net importers include Norway (notably 1990–2020), the United Kingdom, Denmark, the Netherlands and Germany. Recurrent net exporters include countries in Africa and Latin America; some wealthy countries (Canada, Sweden) appear as exporters in particular periods, underscoring complex regional dynamics.

Conversely, the top net exporters are predominantly countries in Africa (Kenya, South Africa), Latin America (Brazil, Mexico, Colombia, Ecuador, Argentina), Canada and Russia in some periods. Most of these countries are sources of specimens that are disproportionately held elsewhere. For example, Kenya appears repeatedly in top-5 exporters across families and periods, Mexico shows net export in multiple periods and Canada curiously appears as both importer and exporter depending on period, suggesting complex temporal dynamics in North American museum activities.

The magnitude and persistence of these imbalances underscore ongoing inequities in specimen custody. While some shifts occur over time — for example, Norway's dramatic emergence as a major importer in 1990-2020 and shifts in the relative ranking of European countries — the overall pattern of concentration in wealthy Northern nations persists. The temporal stability of these patterns, despite decolonisation, capacity-building efforts and policy interventions like the Nagoya Protocol, suggests that structural forces (funding, infrastructure, expertise) continue to drive specimen accumulation in a small set of countries.

**Family‑specific patterns and convergence**

While Felidae shows the most consistent decline in inequality (no recent rebound), Canidae displays a pronounced U‑shape and maintains slightly higher reciprocity in early/mid‑century and Mustelidae shows a mid‑century reciprocity peak with relatively stable inequality. Despite these differences, families converge on three system‑level features by 1990–2020: reduced participation, sharply lower flow complexity and persistently low reciprocity. Correlations amongst nodes, edges and Gini (*r* > 0.85, *p* < 0.001) suggest common drivers across taxa.

## Discussion

Our time‑resolved analysis of specimen flows for Canidae, Felidae and Mustelidae reveals four consistent features of the global geography of natural history collections over the past 120 years: (1) Holdings are highly concentrated in a small set of wealthy nations — most prominently the United States, which holds roughly half of all digitised specimens in our dataset; the top ten holding countries account for ~ 90 %; (2) Inequality trajectories diverged by family: Mustelidae showed a U‑shaped pattern (Gini ~ 0.85 in 1900–1929, declining to ~ 0.77 in 1960–1989, then rebounding sharply to ~ 0.86 in 1990–2020), while Canidae showed a gradual sustained decline (from ~ 0.87 to ~ 0.81) and Felidae exhibited the steepest democratisation (from ~ 0.89 to ~ 0.84); (3) Flow networks contracted from ~ 100–111 countries and ~ 234–262 edges mid‑century to ~ 75–83 countries and ~ 115–160 edges in 1990–2020; (4) Despite this simplification, networks remained highly cohesive (giant component ~ 95–100 %), while reciprocity stayed uniformly low (<~ 0.07), indicating predominantly unidirectional transfers from biodiversity‑rich regions to repository‑rich nations. These patterns are broadly parallel across the three families, suggesting system‑level dynamics rather than taxon‑specific effects.

These patterns show both convergent and divergent dynamics across the three families. Network contraction and persistently low reciprocity are consistent across all families, suggesting system-level changes in the global museum enterprise. However, family-specific inequality trajectories — particularly Mustelidae's sharp re-concentration compared to Canidae and Felidae's sustained democratisation — indicate that taxon-specific factors (e.g. collection priorities, research trends, commercial trade regulations) also shape curatorial patterns. Together, these trends point to persistent structural imbalances in specimen custody and raise important questions about equity, access, capacity and the future of specimen-based biodiversity science.

**Why ~ 30‑year periods**?

Aggregating records into four approximately equal 30‑year periods (last period = 31 years: 1900–1929, 1930–1959, 1960–1989, 1990–2020) improves comparability by holding duration nearly constant, while aligning with recognisable historical phases (colonial era and early expeditions; post‑war expansion; decolonisation and the rise of conservation biology; the digital/ABS era). This framing clarifies that the mid‑ to late‑20^th^ century (especially 1960–1989) marked the apex of globally distributed collecting and exchange and that democratisation of holdings unfolded across multiple decades rather than a short‑lived episode.

**Interpreting recent declines: signal vs. digitisation artefacts**

Lower counts and reduced participation in 1990–2020 plausibly reflect a mix of factors: real changes in collecting and exchange; digitisation lags for recent accessions; and uneven data‑sharing. Recent analyses of global specimen collection trends using GBIF data have documented substantial declines in specimen collecting rates across major taxonomic groups since the mid-20^th^ century, with forecasting models indicating these declines represent genuine reductions in collecting activity rather than solely digitisation artefacts (Forbes et al. 2025). Several observations in our dataset argue that the contraction is not solely an artefact: (i) network structure changes (stable or increasing density coupled with very low reciprocity) suggest reconfiguration, not just missing data; (ii) geographic composition shifts (e.g. Norway's emergence as a large holder) indicate heterogeneous investment and priorities; and (iii) per‑period rates (countries active per year; flows per year) are lower even accounting for duration. We interpret the most recent period as a combination of under‑digitisation of new material and a genuine narrowing of fieldwork breadth and inter‑institutional exchange. Repeating the analysis as coverage improves will help apportion these effects.

It is interesting to note Norway’s prominence as a holding country for our focal families illustrates how collection history and data mobilisation interact. Although Norway is not typically highlighted as a global centre for mammal systematics in the same way as, for example, the United Kingdom, France or Germany – Norwegian institutions maintain substantial Arctic and boreal vertebrate collections, including carnivores acquired through long-term regional field programmes. At the same time, Norway has invested heavily in digitisation and GBIF publication, so that likely a comparatively large fraction of these holdings are visible in our GBIF-derived dataset. By contrast, some historically important mammal collections may remain partially undigitised, available only through non-GBIF portals or lacking complete metadata. Norway’s rise in our networks, therefore, likely reflects a combination of genuine strength in particular faunas (notably mustelids) and high data-mobilisation intensity, underscoring that our results capture the geography of GBIF-visible holdings as much as the geography of physical collections.

**Mid‑century peak, contemporary contraction**

Network size and complexity peaked in 1930–1959 to 1960–1989, consistent with robust post‑war infrastructure, large expeditionary programmes and fewer regulatory constraints. By 1990–2020, participating countries dropped by ~ 25–32 % and edges by ~ 39–56 %. The decline likely reflects interacting drivers: more complex permitting and ABS compliance; stagnant funding for field taxonomy and curation; shifts towards methods less reliant on voucher specimens; and concentration of capacity in a smaller set of well‑resourced institutions. The paradox is stark: even as biodiversity urgency has grown, global participation in specimen‑based work appears to have narrowed.

At the same time, our carnivore-focused dataset does not capture all dimensions of collection growth. Recent syntheses of South American mammal collections show that many institutions in the region have prioritised small-bodied taxa (e.g. non-volant small mammals and associated genetic resources), which are easier to collect and prepare and remain taxonomically challenging, rather than large carnivores (Weksler et al. 2025) . As a result, South American collections may be under-represented in our networks relative to their broader importance for mammalogy and some apparent regional declines in carnivore flows likely coincide with ongoing expansion in other mammal groups.

**Inequality’s “democratisation then rebound**”

Mustelidae shows a pronounced U‑shaped inequality trajectory: mid‑century democratisation (Gini declining from ~ 0.85 to ~ 0.77) followed by sharp re‑concentration in 1990–2020 (rebounding to ~ 0.86). This pattern likely reflects multiple interacting factors. First, regulatory constraints may play a role: many mustelids (otters, sea otters, weasels) are CITES-listed or subject to trade restrictions, potentially creating barriers for range-country specimen export, while consolidated collections in wealthy nations retain historical holdings. Second, taxonomic priorities may drive re-concentration: Mustelidae has undergone extensive phylogenetic revision in recent decades, potentially driving specimen loans or molecular sampling towards institutions with specialised expertise and genomic facilities. Third, ecological and survey challenges may limit range-country capacity building: many mustelids are small, cryptic, low-density species that are difficult to survey and collect and mustelid diversity peaks in regions (Southeast Asia, tropical South America) where museum infrastructure has historically been limited.

In contrast, Canidae and Felidae show declines in inequality (to ~ 0.81 and ~ 0.84, respectively), with Felidae exhibiting the steepest democratisation. Felidae, in particular, may benefit from higher conservation visibility and from targeted investments in range-country research and conservation networks, including the IUCN SSC Cat Specialist Groups, multi-country initiatives, such as the joint Convention on International Trade in Endangered Species (CITES)–Convention on Migratory Species, African Carnivores Initiative and NGO-led programmes (e.g. Panthera’s global wild cat conservation portfolio and related alliances), many of which explicitly invest in local capacity for monitoring and applied research in carnivore range states. The divergent trajectories suggest that sustained range-country investment, targeted collaborations and attention to taxon-specific collection and regulatory dynamics can help counteract the re-concentration pressures observed for Mustelidae.

**Reciprocity remains structurally low**

Across families and periods, reciprocity (bidirectional exchange) is near zero to very low. This points to a persistent asymmetry: specimens predominantly flow from source countries to a small set of repository nations and rarely move back. Low reciprocity concentrates physical access and expertise in holder countries, leaving origin‑country researchers comparatively disadvantaged even when data are digitally visible. Raising reciprocity requires more than open data; it requires institutional commitments to reciprocal loans, distributed reference collections and accession policies that retain shares of series in origin countries where feasible.

**ABS frameworks and legacy imbalance**

Post‑2010 ABS frameworks (e.g. the Nagoya Protocol) govern future access and benefit‑sharing, but do not retroactively address the legacy distribution of specimens — most of which were collected pre‑Nagoya. Our results show that structural imbalances persist in the post‑Nagoya period, likely due to implementation hurdles, capacity asymmetries and the limited scope of ABS to address historical accumulation. More effective equity will pair ABS compliance with capacity building, digital/physical repatriation where appropriate and partnership standards that embed shared benefits and co‑authorship into permitting and funding.

Regulatory frameworks and collaborative initiatives likely interact to produce the family-specific trajectories we observe. Felidae and large Canidae include many CITES-listed and highly regulated species and agreements, such as CITES and the Convention on Biological Diversity, have reshaped how these taxa can be collected, traded and moved across borders. At the same time, these families benefit from extensive conservation networks and capacity-building efforts in range countries, which may help sustain or even expand in-country collections and expertise. By contrast, Mustelidae are dominated by small- to medium-bodied mammals, many of which are less individually high-profile and our results show a partial rebound in inequality for this family. Together, these patterns suggest that recent changes in the geography of holdings reflect not only the constraining effects of regulatory instruments, but also uneven access to collaborative programmes, funding and technical support across taxa and regions.

**Network structure and the problem of low reciprocity**

The extremely low and declining reciprocity in specimen flows is one of our most troubling findings. Reciprocity values below 0.06 in recent periods indicate that fewer than 6% of country-pair relationships involve bidirectional exchange. This pattern reveals specimen flows as fundamentally extractive: biodiversity-rich countries in the Global South predominantly supply specimens to institutions in the Global North, with minimal return flows.

Low reciprocity is incompatible with equitable, collaborative science. True partnerships should involve bidirectional flows of specimens, expertise, capacity and benefits. The current pattern instead resembles colonial-era extractive models, wherein specimens were removed from source countries and concentrated in imperial centres. While legal and ethical frameworks have evolved — notably the Convention on Biological Diversity and Nagoya Protocol — our data suggest that these frameworks have not substantially altered flow patterns. Specimens continue to move overwhelmingly from origin to destination with little reciprocation.

Several structural factors perpetuate low reciprocity: (1) **Institutional capacity**: countries with limited curation infrastructure, expertise and funding may lack the capacity to steward large collections, leading researchers to deposit specimens in better-resourced institutions abroad "for safekeeping"; (2) **Research access**: scientists in museum-rich countries have better physical and intellectual access to specimens, creating self-reinforcing advantages; (3) **Taxonomic expertise**: many taxonomic specialists are based in museums in wealthy nations, requiring specimens to be transferred there for identification or study; (4) **Funding structures**: research grants from wealthy nations often require specimens to be deposited in institutions in the funding country; and (5) **Digital access gaps**: even when specimens remain in source countries, lack of digitisation means they are invisible to global research communities.

**Implications: access, capacity and policy levers**

Digital access (images, 3D, CT, genomics) helps, but can amplify advantages where digitisation capacity is already concentrated. Equitable stewardship requires investments that move beyond data portals to people and places:

- **Institutions (repository nations)**: commit to reciprocal loans and split‑series accession; prioritise digitisation of under‑represented origin regions; fund long‑term training and equipment for partner institutions;
- **Funders**: treat capacity building and digitisation in under‑represented regions as core scientific merit; require equitable partnerships and allow in‑country deposition;
- **Policy-makers**: streamline permits while upholding sovereignty; encourage regional museum networks; include mechanisms for legacy collections in ABS implementation; recognise taxonomy/curation as infrastructure deserving sustained support.

**Limitations and future work**

Results reflect digitised, shared holdings, not complete institutional inventories and GBIF metadata seldom distinguish loans from permanent transfers. Our country-of-origin estimates are based on recorded collection localities and do not systematically distinguish wild-collected material from specimens derived from captive populations (e.g. zoo animals) or from other acquisition pathways (e.g. targeted scientific collecting, opportunistic salvage, trophy hunting, commercial trade). Country-level analyses mask within-country heterogeneity. We focus on three mammal families; patterns may differ for other taxa, particularly for non-carnivore small mammals, invertebrates, plants and other groups that are less affected by CITES listings and trophy-hunting dynamics. Priorities for future work include: repeating the analysis as coverage improves (especially for 1990–2020), applying the same framework to additional taxonomic groups with different regulatory and life-history profiles, incorporating loan/repatriation metadata and digitisation timestamps, analysing institution-level dynamics, linking flows to authorship/outputs and evaluating policy impacts with quasi-experimental designs.

As our workflow is taxon-agnostic, the same approach can be applied to additional groups, including less-regulated taxa, such as non-carnivore small mammals, invertebrates and plants, to assess how general these patterns are and how regulatory policies and collaborative agreements jointly shape the contemporary geography of collections.

## Conclusions

The global geography of specimen custody is neither static nor inevitable. Our analysis reveals a system that peaked mid-century, partially democratised, then diverged: Mustelidae re-concentrated sharply to near-initial inequality levels, while Canidae and Felidae sustained gradual democratisation. Simultaneously, specimen collection networks contracted dramatically — fewer countries, fewer flows, persistently low reciprocity — a decline now documented to reflect genuine reductions in collecting activity, not merely digitisation lags (Forbes et al. 2025). Yet, Felidae's sustained decline in inequality and Norway's recent emergence as a major holder demonstrate that investment and strategic choices can reshape outcomes. Achieving equitable stewardship — where holdings, expertise and capacity align with biodiversity — requires sustained action across institutions, funding systems and policy. As global specimen collecting erodes at precisely the moment when climate change and biodiversity loss demand comprehensive spatiotemporal data, this baseline provides both a benchmark for progress and an urgent call to action.

## Supplementary Material

3B6427D0-065F-54A3-A232-5D32E9AD9AA3 10.3897/BDJ.14.e185833.suppl1 Supplementary material 1 Supplementary Figure 1 Data type Figure Brief description Canidae specimen flow networks across time periods. Four network maps showing country-to-country flows for Canidae across 30-year periods (1900-1929, 1930-1959, 1960-1989, 1990-2020). Canidae networks show peak complexity in 1930-1959 and 1960-1989 (207-235 flows), then a decline to 144 flows in 1990-2020 (39% reduction from peak). The United States dominates as a central hub throughout all periods. Canidae exhibits a U-shaped Gini trajectory (0.89 → 0.77 → 0.84) and moderate reciprocity (0.07-0.11 declining to 0.08). File: oo_1510345.png https://binary.pensoft.net/file/1510345 Mills

3A0E9F28-B92D-5DC4-9CB5-C4B01DCD73A6 10.3897/BDJ.14.e185833.suppl2 Supplementary material 2 Supplementary Fig.2 Data type Figure Brief description Felidae specimen flow networks across time periods. Four network maps for Felidae. Felidae shows flows ranging from 212 in 1900-1929, peaking at 245 in 1960-1989, then collapsing to 115 in 1990-2020 (56% decline — steepest reduction amongst the three families). United States dominates the largest hub throughout. Felidae exhibits the steepest inequality decline without a rebound (0.89 → 0.81 linear), the lowest reciprocity across all periods (< 0.04) and the most dramatic network contraction, likely reflecting high conservation profile and CITES restrictions. File: oo_1510348.png https://binary.pensoft.net/file/1510348 Mills

6E9F86A6-5757-5D4A-9DED-F47B92BF1226 10.3897/BDJ.14.e185833.suppl3 Supplementary material 3 Supplementary Fig. 3 Data type Figure Brief description Mustelidae specimen flow networks across time periods. Four network maps for Mustelidae. Mustelidae shows highest flow complexity of the three families: 234 flows in 1900-1929, peaking at 262 in 1960-1989, declining to 160 in 1990-2020 (39% reduction). Scandinavia is prominent with Norway, Sweden, Denmark, Finland as substantial hubs. Mustelidae exhibits a distinctive 1930-1959 reciprocity peak (0.15—highest in the entire dataset), likely reflecting mid-century institutional exchange programmes driven by fur-trade economic importance, though reciprocity declines to 0.08 by 1990-2020. File: oo_1510349.png https://binary.pensoft.net/file/1510349 Mills

## Figures and Tables

**Table 1.** Summary of specimen flow networks by temporal period. Values represent ranges across three mammal families (Canidae, Felidae, Mustelidae) unless otherwise noted. Network size = number of countries participating in international specimen flows (nodes); Complexity = number of unique directed country-to-country specimen flows (edges); Gini coefficient measures inequality in specimen holdings (0 = perfect equality, 1 = perfect inequality); Reciprocity = proportion of country pairs showing bidirectional exchange; Giant component = percentage of countries in largest connected component. All metrics exclude within-country flows (self-loops).

**Metric**	**1900–1929**	**1930–1959**	**1960–1989**	**1990–2020**
**Network size (countries)**	100–111	100–111	105–110	75–84
**Network complexity (flows)**	207–240	234–262	209–245	115–160
**Gini coefficient (inequality)**	0.85–0.89	0.85–0.87	0.77–0.89	0.81–0.86
**Reciprocity**	0.01–0.11	0.05–0.15	0.04–0.11	0.04–0.08
**Giant component (%)**	95–100	95–100	95–100	95–100

**Dataset totals (1900–2020)**:

Total specimens: 253,131 - Canidae: 94,401 (37%), Felidae: 41,113 (16%), Mustelidae: 117,617 (46%)

Geographic concentration: Top 10 holding countries account for ~ 89-90% of all specimens. United States holds ~ 50% of specimens across all families and periods. Temporal trends: Network size declined 23–29% from mid-century peak to 1990–2020. Network complexity declined 39–56% over the same period. Mean reciprocity across all periods: 0.075 (7.5%), indicating predominantly unidirectional flows.
